# Combining Ecological Momentary Assessment and Social Network Analysis to Study Youth Physical Activity and Environmental Influences: Protocol for a Mixed Methods Feasibility Study

**DOI:** 10.2196/68667

**Published:** 2025-02-21

**Authors:** Tyler Prochnow, Genevieve F Dunton, Kayla de la Haye, Keshia M Pollack Porter, Chanam Lee

**Affiliations:** 1 Department of Health Behavior School of Public Health Texas A&M University College Station, TX United States; 2 Department of Preventive Medicine Keck School of Medicine University of Southern California Los Angeles, CA United States; 3 Department of Psychology Center for Economic and Social Research University of Southern California Los Angeles, CA United States; 4 Department of Health Policy and Management Bloomberg School of Public Health Johns Hopkins University Baltimore, MD United States; 5 Department of Landscape Architecture & Urban Planning College of Architecture Texas A&M University College Station, TX United States

**Keywords:** physical activity, youth, social environment, built environment, ecological momentary assessment, social network analysis, phenotypes, accelerometry, GPS

## Abstract

**Background:**

Physical activity (PA) is crucial for youth health, but up to 74% of adolescents fail to meet recommended levels, especially during summer when structured supports associated with school are not available. The social and built environments significantly influence youth PA; yet, their complex interactions remain poorly understood. This study aims to evaluate the feasibility of combining ecological momentary assessment (EMA) and social network analysis to examine bidirectional influences among youth PA, built environments, and social networks during summer.

**Objective:**

The objectives are to (1) evaluate the feasibility and acceptability of the combined EMA and Social Network Analysis protocol, and (2) identify phenotypes using person-level, microtemporal, and dynamic overlap between social and built environments.

**Methods:**

This mixed methods feasibility study with an exploratory observational component will recruit 120 youth aged 12 years to 15 years from an urban school district in Central Texas, US. Participants will first complete a baseline survey to report their general social network patterns and environmental perceptions. Then participants will wear an ActiGraph LEAP accelerometer and respond to EMA prompts via smartphone for 7 days. EMA will assess real-time perceptions of social networks and surrounding built environments, which will be time-matched with accelerometer-assessed PA data. GPS coordinates will be collected with each EMA prompt to assess features of the built environment. Follow-up semistructured interviews will assess protocol acceptability.

**Results:**

This study has been funded by the National Heart, Lung, and Blood Institute. Data collection is expected in the summers of 2025, 2026, and 2027.

**Conclusions:**

This innovative approach combines EMA, SNA, accelerometry, and GPS data to provide unprecedented insights into the dynamic interplay between social networks, built environments, and youth PA during summer. Findings will inform the development of more targeted, effective interventions to promote PA among youth. While limitations include potential participant burden and generalizability, the study’s strengths in capturing real-time, contextualized data make it a valuable contribution to understanding youth PA determinants.

**International Registered Report Identifier (IRRID):**

PRR1-10.2196/68667

## Introduction

### Background

Physical activity (PA) is crucial for youth health and development, offering a wide range of physical, psychological, and social benefits [1]. However, roughly 74% of young people fail to meet recommended PA levels [2], putting them at risk for various health issues both in the short and long term [1]. Summer presents a unique challenge for youth PA, as it represents a significant departure from the social and built environment supports typically offered by schools [3,4]. During the academic year, schools provide structured physical education classes, organized sports, and supervised recess periods, all of which contribute to youth PA levels [3,4]. The absence of these supports during summer leads to substantial changes and variability in how youth experience and are influenced by their social and built environments [5,6].

The social and built environments have been identified as key modifiable factors influencing youth PA, with research suggesting complex and nuanced relationships between these environmental factors and PA behaviors [7]. The social environment encompasses interpersonal relationships, social networks, and community norms, while the built environment includes physical structures, facilities, and design elements that can either facilitate or hinder PA. Despite growing research in this area, our understanding of how different layers of the social ecological model impact youth PA remains unclear, particularly during the summer months when the structured supports provided by schools are absent [8,9]. This knowledge gap hinders the development of effective interventions to promote PA among youth, especially during critical periods like summer. Moreover, there is considerable variability between youth in terms of the social and built environment factors they are exposed to during the summer months, as well as the impact these factors have on their PA levels [3,4,8]. For instance, some youth may be more affected by built environment barriers (eg, lack of safe parks or playgrounds) despite having a supportive social environment (eg, friends who enjoy physical activities), while others may face disparities in both domains [1,10,11]. This heterogeneity in experiences and influences underscores the need for more sophisticated research approaches that can capture and analyze these complex relationships.

Recent reviews of existing evidence highlight a significant need for more nuanced measurement approaches, including the use of intensive longitudinal data (ILD) to understand the complex variability (both within and between-subject) as well as the independent and mutual effects of social and built environments on youth PA [7,12,13]. Traditional cross-sectional or even longitudinal studies with infrequent measurement points may fail to capture the dynamic nature of these relationships and the day-to-day variations in youth PA behaviors [12,13]. ILD, on the other hand, allows for the collection of frequent, real-time data that can reveal patterns and associations that might otherwise be missed [12,13]. One promising approach to capturing this variability is through the identification of “phenotypes,” individual-specific webs of links between social and built environmental determinants [14,15]. These phenotypes have the potential to identify overlaps in ILD and salient intervention targets for health behavior [14-16]. By understanding these phenotypes, researchers and practitioners may be able to develop more personalized and effective interventions to promote PA among youth, considering the unique combination of social and built environment factors that influence an individual’s behavior.

Ecological momentary assessment (EMA) has emerged as a valuable method for collecting ILD by gathering real-time self-reports of behaviors, contexts, and perceptions in naturalistic settings [12,13]. EMA typically involves prompting participants multiple times throughout the day to report on their current activities, feelings, and surroundings, providing a rich, contextual dataset that captures the ebb and flow of daily life. This approach offers several advantages over traditional retrospective self-report measures, including reduced recall bias and the ability to capture within-person variability over time [12,13]. However, previous EMA measures of the social environment have been limited to the presence of others or coparticipation in PA, which may not fully capture the complexity of social influences on youth PA [7,13,17]. Social network analysis (SNA) offers the potential to provide more nuanced information, such as dyadic and personal network measures of social bridging, bonding, norms, and influence [17,18]. SNA allows researchers to map and analyze the structure and composition of an individual’s social network, providing insights into how social connections and interactions may influence PA behaviors. By combining EMA and SNA approaches, researchers may be able to gain a more comprehensive understanding of the dynamic interplay between social factors and PA in youth’s daily lives.

To examine these influences on a more granular level, this project aims to evaluate the feasibility of combining EMA and SNA techniques to collect ILD describing social and built environment associations with youth PA during the summer. This innovative combination of EMA and SNA techniques represents a data-intensive approach, necessitating an evaluation of its feasibility and acceptability in terms of validity, reliability, and respondent burden. The integration of these methods has the potential to provide unprecedented insights into the complex relationships between social networks, built environments, and PA behaviors among youth. For example, EMA data may show a youth’s PA spikes when they are with a specific friend, but only if they are within a half-mile radius of a park, as indicated by GPS data. SNA reveals this friend is not particularly active themselves, but rather may be influential in triggering PA in groups, potentially exposing the youth to varied activity opportunities. This complex interplay suggests that interventions targeting both social network dynamics and built environment access could be more effective than addressing either in isolation, highlighting the value of integrating EMA and SNA approaches to understand youth PA patterns.

This complex framework also presents challenges in terms of data collection, participant compliance, and analytical complexity. The intensive nature of the EMA protocol, combined with the detailed social network data collection, may lead to significant participant burden, potentially resulting in missing data, reduced compliance over time, or even selective attrition of certain participant subgroups. Moreover, the frequent prompts and awareness of being monitored could introduce reactivity, where participants alter their behavior or reporting patterns, while the complexity of the data collected across multiple platforms (EMA, GPS, and accelerometry) presents substantial challenges in data integration, cleaning, and analysis, requiring sophisticated statistical approaches to handle the multilevel, time-varying nature of the data. By conducting a feasibility study, researchers can identify potential barriers and refine the methodology before implementing it on a larger scale. This study seeks to address the critical need for more comprehensive and nuanced understanding of the complex interplay between social and built environments and their impact on youth PA during the summer months. The findings from this research could inform the development of more effective, targeted just in time adaptive interventions to promote PA among youth, ultimately contributing to improved health outcomes and reduced health disparities in this population.

### Study Aims

The study has 2 primary aims. Aim 1 is to evaluate the feasibility and acceptability of an EMA protocol that combines the collection of social network characteristics using SNA and built environment characteristics corresponding to PA among youth during the summer. This aim will assess metrics such as EMA response rates, accelerometer wear time, and qualitative feedback from participants to determine the viability of the combined EMA-SNA approach. Aim 2, which is exploratory, seeks to identify and classify phenotypes by using microtemporal dynamic overlaps between social and built environments. Aim 2 will examine these phenotypes and their association with PA among youth in summer. These aims will use cluster analysis to identify subgroups of social and built environment patterns within the sample followed by dynamic structural equation modeling (DSEM) to analyze the bidirectional dynamics of social and built environment factors and PA [19,20]. These aims will provide a comprehensive assessment of the proposed methodology’s feasibility and potential for uncovering nuanced relationships between environmental factors and youth PA during summer.

## Methods

### Study Design

This study will use a mixed methods approach to evaluate the feasibility and acceptability of combining EMA and SNA techniques for assessing social and built environment influences on youth PA during summer. The research design involves recruiting 120 youth aged 12 years to 15 years (entering the seventh to the ninth grades) from a local school district. An initial cohort of 20 youths will be recruited in year 1 of the project to assess initial feasibility and pilot the measures. Participants will be divided into 2 cohorts (n=50 each) across years 2 and 3, with each cohort further split into 5 groups (n=10) to facilitate data collection throughout the summer.

### Conceptual Framework

The conceptual model guiding this study is grounded in a social ecological model, recognizing that youth PA is influenced by multiple, interacting layers of environmental factors [21,22]. At its core, the model posits that youth PA is shaped by a dynamic interplay between social and built environment elements, with each exerting both independent and synergistic effects on PA behaviors [7,23]. The social environment component encompasses the structure and quality of social networks, including aspects such as social bridging, bonding, norms, and influence [17,18,24]. These social factors are theorized to provide support, motivation, and opportunities for PA, but may also act as barriers in some contexts [25,26]. The built environment element includes physical structures, facilities, and urban design features that can either facilitate or hinder PA [27,28]. This includes factors such as neighborhood walkability, access to recreational spaces, and perceived safety [29-31].

Critically, the model emphasizes the reciprocal nature of these relationships, acknowledging that youth’s PA behaviors can, in turn, influence their perceptions and interactions with both social and built environments [32,33]. This bidirectional conceptualization allows for a more nuanced understanding of how environmental factors and PA behaviors coevolve [7]. The model also incorporates the concept of phenotypes, representing individual-specific patterns of associations between social and built environment factors and PA [14,34]. These phenotypes are theorized to capture the heterogeneity in how youth respond to and interact with their environments, reflecting the complex, person-specific nature of PA determinants [35,36]. By integrating these various components, the conceptual model provides a comprehensive framework for understanding the multifaceted influences on youth PA, particularly during the unique context of summer months when typical school-based structures are absent [8,9].

### Participants and Procedures

The study will recruit 120 youth participants aged 12 years to 15 years (entering the seventh to ninth grades) from a local school district. This age range was selected based on previous research indicating significant declines in PA and increases in peer influences during this developmental period [37,38]. Recruitment will be stratified to ensure equal numbers across sex and grade levels. Participant recruitment will occur through a partnership with a school district in central Texas, United States, which serves a diverse student population. The district-wide demographics include 72% of students classified as being at risk of dropping out of school and 77% eligible for free or reduced-price lunch. The student population is approximately 60% Hispanic or Latinx, 18% Black or African American, and 19% White. Recruitment will occur in 2 phases. First, a feasibility and pilot cohort (n=20) will be recruited in year 1. Next, 2 additional cohorts (n=50 each) will be recruited over years 2 and 3 to manage researcher burden and resource allocation. Each cohort will be further divided into 5 groups (n=10) to facilitate data collection throughout the summer with 1 group participating in data collection each week. Participants will be asked to provide a list of weeks that they would be able to participate in the program to avoid scheduled family vacations and other activities that would alter their normal activity level. Participants will then be assigned a week at random from their available weeks. An informational sheet will be sent to eligible youth and their guardians in cooperation with the school district.

The study protocol consists of three main components: (1) an initial survey, (2) a 7-day EMA period, and (3) a follow-up interview. During the initial study visit, participants will complete researcher-administered surveys assessing their personal social networks and perceptions of the built environment around their home. They will also receive training on the use of the EMA application and the ActiGraph LEAP accelerometer. For the 7-day EMA period, participants will wear the ActiGraph LEAP accelerometer on their nondominant wrist 24 hours per day. They will respond to EMA prompts via a smartphone application 6 times daily over 12 hours [39]. The application (LifeData) will be downloaded onto their personal phones (Android or Apple). If the adolescent does not have a phone, a research device will be provided with all other functionality disabled. Prompts will be delivered at random during six 2-hour windows across the day with no prompt being sent within 30 minutes of the previous prompt. The prompting schedule will be adjusted to accommodate each participant’s sleep and wake schedule, with 3 options available (8 AM to 8 PM; 9 AM to 9 PM; and 10 AM to 10 PM). Each prompt will include short questionnaires (2-3 mins) assessing momentary perceptions of social and built environments. Following the 7-day EMA period, participants will return for a follow-up visit to return the equipment and participate in a qualitative interview. This interview will assess the acceptability of the EMA and accelerometry protocols and gather additional contextual information about their experiences during the study period.

### Survey Variables

The survey component of this study will assess various aspects of the social and built environments and demographic information. These measures are collected during the initial study visit and provide baseline data for each participant.

### Demographic Information

Participants will provide demographic information, including age, sex, race, ethnicity, household income (reported by parents or guardians), parental education level, and home address (for Geographic Information System analysis). These data will be used to characterize the study sample and explore potential moderating effects on the relationships between social networks, built environments, and PA.

### Social Network Characteristics

To assess social network characteristics, participants will be asked to list up to 10 people (also termed alter) they interacted with most in person over the last 7 days, a method previously used in personal network research [17,18,24]. For each person listed, participants will provide information on relationship type, frequency of contact, perceived frequency of the alter’s PA, frequency of coparticipation in PA, perceived closeness, and likelihood of joining the alter in new activities or PA. Participants will also report if each pair of alters knows each other, allowing for the analysis of network structure [17,18]. These data will be used to calculate various social network measures, including social bridging (network size, effective size, and diversity), social bonding (density, proportion of frequent contact, and mean closeness of connection), social norms (frequency of coparticipation in PA and mean perception of alter PA), and social influence (proportion of likely influencers and mean suggestibility) [17,18,40]. In addition, individuals listed in this survey will also be included in EMA prompts as potential responses to items regarding the social environment. In this manner, SNA alters perceptions can be combined with EMA prompt answers to offer opportunities to connect the data set and better explain the social environment as collected by the EMA prompts.

#### Built Environment Perceptions

To assess built environment perceptions, the Neighborhood Environment Walkability Scale–Youth (NEWS-Y) will be used [31]. This scale, adapted from the adult version and validated for use with youth aged 12 years to 18 years, assesses several domains of neighborhood walkability including PA resource access, land-use mix, walkability, neighborhood aesthetics, safety (crime and traffic), walking and bicycling facilities, street connectivity, and residential density [29]. These measures provide a comprehensive assessment of youth perceptions of their neighborhood-built environment.

#### EMA Items

The EMA protocol is designed to capture real-time data on participants’ social context, built environment perceptions, and PA. EMA prompts will be delivered 6 times per day over a 12-hour period for 7 consecutive days, using a smartphone application [41,42]. Prompts will be delivered at random during six 2-hour windows across the day with no prompt being sent within 30 minutes of the previous prompt. Each EMA prompt is designed to be completed in 2-3 minutes to minimize participant burden while still capturing key variables of interest. The combination of these EMA items with the continuous accelerometer data and GPS coordinates will provide a rich, contextualized dataset for examining the dynamic relationships between social and built environments and youth PA during summer [36,42]. The specific items included in the EMA prompts are in Table 1.

**Table 1:** Domains, ecological momentary assessment prompts, and response options.

Domain	Ecological momentary assessment prompt	Response option
Current activity	“What were you doing right before the beep went off [Choose your main activity]?”	Reading/computer/phone, watching TV/movies, eating/drinking, physical activity/exercising, socializing/hanging out, and others.
Social context	“WHO were you with just before the beep went off?”	Free response for person(s) listed in the network, other friends, siblings, parents, other family members, others, or people they did not know. (Select all that apply)
Social bridging	N/A^a^	Measured by the diversity of social context and frequency of persons not known.
Social bonding	“How close (emotionally) do you feel to those around you at this moment?”	Visual analog scale anchored from “not at all” to “extremely close”
Social norms	“How physically active do you think the people around you are normally?”	Visual analog scale anchored from “not at all” to “extremely active”
Social influence	“If someone you are with suggested doing something physically active, how likely would you be to join?”	Visual analog scale anchored from “not at all” to “extremely likely”
Built context	“Where were you just before the beep went off?”	Home (indoors), home (outdoors), care program (indoors), outdoors (not at home), car/van/truck, and other
Safety	“How safe do you feel in the current setting?”	Visual analog scale anchored from “not at all” to “extremely”
Pleasantness	“How pleasant is the physical setting?”	Visual analog scale anchored from “not at all” to “extremely”
Space to be active	“How much space is there to be physically active where you are right now?”	Visual analog scale anchored from “none” to “a lot”
Affective and feeling states	“Right now, how SAD do you feel?” “Right now, how HAPPY do you feel?” “Right now, how FATIGUED do you feel?” “Right now, how ENERGETIC do you feel?” “Right now, how RELAXED do you feel?” “Right now, how TENSE do you feel?” “Right now, how STRESSED do you feel?” “Right now, how FRUSTRATED do you feel?” “Right now, how NERVOUS do you feel?”	Visual analog scale anchored from “not at all” to “extremely”
Interesting or engaging	“What is the most interesting/engaging part of the surrounding environment to you right now?”	Free response

^a^N/A: Not available.

#### Activity Level

Participants will report their current activity at the time of each prompt. Options will include sedentary activities (eg, sitting and lying down), light activities (eg, standing and walking slowly), moderate activities (eg, brisk walking), and vigorous activities (eg, running and sports). This item has been validated against accelerometer measures in previous youth studies [43].

#### Social Context

To assess momentary social context, participants will respond to questions about who they are with now, the number of people present, how close (emotionally) they feel, their perception of how active those around them are being, and their perception of susceptibility to PA suggestions. Previously mentioned individuals from the initial SNA survey will be provided as response options in these prompts to further understand social influence and norms. These items are designed to capture both the presence of others, social norms related to PA, and susceptibility to influence in the immediate environment.

#### Built Environment Perceptions

Participants will be asked about their current physical location and their perceptions of the immediate built environment. Questions will address the type of location, perceived safety of the current environment, pleasantness of the surroundings, and availability of space to be physically active. These items are adapted from previous EMA studies on built environment perceptions and PA [36,41].

#### Mood and Motivation

To capture psychological factors that may influence PA, participants will be asked about their current mood (using a brief affect scale). These items allow for the examination of how momentary psychological states may interact with social and built environment factors to influence PA [41].

### Qualitative Inquiry

To complement the quantitative data collected through surveys, EMA, and accelerometry, this study incorporates a qualitative component to gain deeper insights into participants’ experiences and perceptions. Following the 7-day EMA period, semistructured interviews (Multimedia Appendix 1) will be conducted with all feasibility participants (n=20). These interviews will serve multiple purposes: (1) to assess the feasibility and acceptability of the EMA and accelerometry protocols; (2) to gather contextual information about participants’ experiences during the study period, such as whether they encountered difficulties answering prompts while engaged in physical activities, if the device interfered with their sleep, or if they felt compelled to alter their behavior due to being monitored; and (3) to explore any anomalies or patterns observed in the quantitative data. The interviews will probe participants’ thoughts on answering EMA questions in real time, any privacy concerns they may have had, and their overall experience with the study protocol. In addition, an adapted version of the System Usability Scale will be integrated into the interview to further assess the acceptability and usability of the protocol [44]. These interviews will be audio-recorded, transcribed verbatim, and analyzed using thematic analysis to identify key themes related to the study’s feasibility.

#### Analysis Plan for Feasibility Assessment (Aim 1)

The feasibility assessment will examine multiple quantitative metrics including EMA response rates, with a target of at least 70% completion, accelerometer wear time aiming for 5 or more valid days with more than 10 hours per day, and participant retention rates targeting 80% or higher. We will calculate descriptive statistics including means, SD, and 95% CIs for these metrics. In addition, we will assess patterns of missing data and examine whether compliance varies systematically by participant characteristics using logistic regression models.

#### Qualitative Analysis Approach

The qualitative analysis will follow the Braun and Clarke [45,46] reflexive thematic analysis framework, which emphasizes the active role of researchers in identifying patterns of meaning across the dataset. The analysis process will begin with data familiarization, during which 2 trained researchers will independently immerse themselves in the data through repeated reading of interview transcripts. This will be followed by systematic initial coding using NVivo (Lumivero) software, with researchers generating codes across the entire dataset and paying particular attention to data related to protocol feasibility and acceptability. The researchers will then develop themes by sorting codes into meaningful patterns and creating a preliminary thematic framework that captures key aspects of participants’ experiences. These themes will undergo refinement through review in relation to both coded extracts and the full dataset to ensure they form coherent patterns and accurately represent the data. Clear definitions will be developed for each theme to identify the essence of what each captures about participants’ experiences with the protocol. The final analysis will be synthesized into a coherent narrative, supported by illustrative quotes.

#### Ensuring Qualitative Rigor

To enhance the trustworthiness of our findings, we will implement several complementary strategies [45,47]. For credibility, we will use of investigator triangulation, with multiple researchers independently coding data and comparing interpretations, along with member checking conducted with a subset of participants to verify our interpretations reflect their experiences. To establish dependability, we will maintain a detailed audit trail documenting analytical decisions and theoretical development throughout the analysis process. Confirmability will be addressed through researchers’ engagement in reflexive journaling to document their positionality and potential biases, with regular team meetings including discussions of how researchers’ backgrounds and perspectives might influence interpretation. For transferability, we will provide rich, detailed descriptions of the study context and participant characteristics to allow readers to assess the applicability of findings to other settings.

#### Integration of Quantitative and Qualitative Findings

The mixed methods analysis will use a convergent parallel design where quantitative and qualitative data are analyzed separately and then merged to provide a comprehensive understanding of protocol feasibility [48]. Areas of convergence and divergence between quantitative metrics and qualitative experiences will be explicitly examined and discussed. This integrated analysis will inform decisions about protocol modifications and provide crucial insights for implementing these methods in larger-scale studies. The combination of rigorous quantitative metrics with rich qualitative insights will allow us to not only assess the technical feasibility of our protocol but also understand the lived experiences of participants engaging with these novel data collection methods [48].

### Analysis Plan (Aim 2)

To address the challenge of integrating data collected at different frequencies and levels of analysis, a multistep data processing approach will be used. Initially, accelerometer and GPS data will be aligned using timestamp information, creating a continuous stream of location-tagged PA data. For each EMA prompt, a time window of more than 30 minutes will be established, extracting the corresponding accelerometer and GPS data. This window allows for the capture of PA and location information immediately before and after each self-report, providing context for the EMA responses. Social network data from the initial survey will be treated as a stable characteristic for each participant throughout the study period, with key network metrics (eg, network size and density) linked to each EMA prompt. To account for potential temporal dynamics in social networks, EMA items assessing momentary social context will be used to create time-varying indicators of social influence. The resulting dataset will be structured hierarchically, with EMA prompts nested within days, and nested within participants. This approach allows for the examination of associations between social network characteristics, built environment features, and PA at various temporal scales, from momentary fluctuations to day-level patterns, while accounting for the different data collection frequencies and levels of analysis.

The analysis plan for this study incorporates a range of statistical techniques to address the research aims and account for the complex, multilevel nature of the data. For aim 2, which seeks to identify within-day phenotypes, a multistep analytical approach will be used. Associations will be explored at the hour, day, and person levels to determine which temporal associations are strongest. First, intraclass correlation coefficients (ICCs) will be calculated to estimate the impact of clustering (occasions within days, days within individuals) [49]. DSEM will then be used to analyze the within-person time-series data, accounting for the intensive longitudinal nature of the data and examining the bi-directional effects of social and built environments and PA [19]. DSEM allows for the decomposition of variance in PA into within-person and between-person components, estimating autoregressive parameters and cross-lagged parameters. To identify phenotypes, within-person standardized model estimates from the DSEM model will be used in an individual-level cluster analysis, using Ward’s Method with Euclidean distance for hierarchical agglomerative clustering [20]. Validity of the clusters will be assessed using ANOVAs and chi-squared tests to examine univariate differences between clusters in terms of PA. Sensitivity analyses will be conducted with varying clustering methods, item rescaling, and time points included to assess the robustness of the cluster profiling [50]. Throughout the analysis, considerations will be made for potential sex and age differences, with exploratory moderation tests conducted. Missing data will be handled using maximum likelihood estimation in the multilevel models. All analyses will be conducted using appropriate statistical software packages, with a significance level set at *P*<.05, adjusted for multiple comparisons where necessary using the Bonferroni correction.

### Sample Size Justification and Power Analysis

Our sample size determination was guided by multiple considerations. For aim 1 (feasibility and acceptability), the planned sample size of 20 participants aligns with recommendations for feasibility studies [51]. We will recruit 20 participants in year 1 for initial protocol testing, followed by 2 cohorts of 50 participants each in years 2 and 3. This sample size will allow us to estimate compliance rates with a margin of error of ±9% (assuming 95% CIs), which is adequate for assessing protocol feasibility. For aim 2 (identifying phenotypes), we conducted power analyses using Monte Carlo simulations for DSEM. Based on these simulations, assuming moderate effect sizes (β=0.3) and ICCs of 0.2, a sample of 100 participants providing 42 observations each (6 prompts per day for 7 days) will provide 80% power to detect significant cross-lagged effects at α=.05.

While the study is primarily powered for overall effects, we acknowledge limitations in detecting sex differences. Given our balanced recruitment by sex (50 participants per group), post-hoc power analyses indicate we would only be able to detect large effect size differences (*d*>0.7) between males and females with 80% power. However, previous research in youth PA suggests that while absolute activity levels may differ by sex, the underlying mechanisms of social and built environment influences are generally consistent across sexes [52]. Therefore, while we will explore potential sex differences as secondary analyses, our primary focus is on identifying overall patterns and phenotypes that can inform future intervention development.

### Ethical Considerations

This study was approved by the institutional review board at Texas A&M University (STUDY2024-0473). The study will be performed in accordance with the ethical standards as laid down in the 1964 Declaration of Helsinki and its later amendments. All participants will be asked to provide assent to participate. In addition, guardian permission will also be required for all participants. Data will be deidentified using unique study IDs to protect participants privacy. Participants will receive compensation for their involvement, with a tiered incentive structure to encourage compliance. They will receive US $25 gift cards for the initial survey, return devices after the EMA protocol, and participate in qualitative interviews. An additional US $25 will be provided for meeting compliance benchmarks of at least 5 days of valid accelerometer data (more than 10 hours/day) and at least a 70% response rate for EMA prompts, for a potential total of US $100 throughout the study.

## Results

The study received funding from the National Heart Lung and Blood Institute in May 2024. Data collection is expected to occur during the summers of 2025, 2026, and 2027. Findings are expected to be published in the fall of 2027.

## Discussion

### Principal Findings

This study will yield several important findings. First, regarding aim 1, we expect to demonstrate the feasibility and acceptability of combining EMA and SNA techniques to assess social and built environment influences on youth PA during summer. We also expect to identify potential challenges and areas for improvement in the protocol, which will be valuable for future studies using these methods. For aim 2, we anticipate identifying distinct phenotypes that represent different patterns of associations between social and built environment factors and PA. These phenotypes may reveal subgroups of youth who are more or less influenced by specific social or built environment characteristics. For example, we may find a subgroup for whom social network factors are particularly influential on PA, and another for whom built environment features play a more prominent role [7]. We expect these phenotypes to provide insights into the heterogeneity of environmental influences on youth PA, potentially informing more targeted and personalized intervention strategies.

### Strengths

This study has several notable strengths. The combination of EMA and SNA techniques represents an innovative approach to capturing the dynamic interplay between social and built environments and youth PA. This method allows for the collection of rich, contextual data in real-time, reducing recall bias and capturing within-person variability [12]. The use of objective PA measures through accelerometry, coupled with GPS data, provides a comprehensive picture of youth PA patterns and their environmental contexts [53]. In addition, the mixed-methods approach, incorporating both quantitative and qualitative data, allows for a more nuanced understanding of youth experiences and perceptions. The focus on the summer period addresses an important gap in the literature, as this is a time when youth PA patterns may differ significantly from the school year [8]. Finally, the application of advanced analytical techniques such as DSEM and phenotyping represents a cutting-edge approach to understanding the complex, individual-specific nature of environmental influences on PA [14,19].

### Limitations

Despite its strengths, this study has limitations that should be acknowledged. The sample size, while appropriate for a feasibility study, may limit the generalizability of findings, particularly in the identification of phenotypes. The focus on a single geographic area may also limit generalizability to youth in other regions with different social and built environment characteristics. While our sample size is appropriate for our primary aims, we acknowledge limited power to detect sex differences in environmental influence patterns. However, existing literature suggests that fundamental mechanisms of social and built environment influences on physical activity are largely consistent across sexes during early adolescence, even though absolute activity levels may differ. Future larger-scale studies may be needed to fully explore potential sex-specific patterns in these relationships. The intensive nature of the EMA protocol, while providing rich data, may introduce participant burden and potentially affect compliance rates or typical behavior patterns. There is also a possibility of reactivity, where participants may alter their behavior due to awareness of being monitored. In addition, while the study captures a week of data for each participant, this may not represent typical summer PA patterns, which could vary over the course of the entire summer. Finally, while the combination of EMA and SNA provides detailed information on social networks and momentary social contexts, it may not capture all relevant aspects of social influence on PA. Similarly, the built environment measures, while comprehensive, may not account for all relevant features that influence youth PA.

### Conclusions

This study represents an important step forward in understanding the complex interplay between social and built environments and youth PA during summer. By employing innovative methodologies and advanced analytical techniques, we expect to gain unprecedented insights into the dynamic, individual-specific nature of these relationships. The findings from this study have the potential to inform more targeted and effective interventions to promote PA among youth, particularly during the critical summer months. While limitations exist, they are outweighed by the strengths of this study’s design and its focus on an understudied time period makes it a valuable contribution to the field. Future research can build upon these methods and findings to further elucidate the multifaceted influences on youth PA and develop strategies to promote active, healthy lifestyles among diverse youth populations.

## Appendix

Multimedia Appendix 1 Qualitative questions.

## Abbreviations

DSEM: dynamic structural equation modeling
EMA: ecological momentary assessment
ICC: intraclass correlation coefficient
ILD: intensive longitudinal data
NEWS-Y: Neighborhood Environment Walkability Scale–Youth
PA: physical activity
SNA: social network analysis
