# Supplementary material for: Combining Ecological Momentary Assessment and Social Network Analysis to Study Youth Physical Activity and Environmental Influences: Protocol for a Mixed Methods Feasibility Study
Source: JMIR Res Protoc. 2025 Feb 21;14:e68667. doi: 10.2196/68667 (PMC11890139; doi:10.2196/68667)
Supplement: Multimedia Appendix 1 [file resprot_v14i1e68667_app1.pdf]

Qualitative interview guiding questions

1. Can you tell me about your typical day during the past week when you were participating in the study? Were there any unusual events or activities that might have affected your responses?
2. How did you feel about the frequency of the EMA prompts? Did you find them burdensome or disruptive to your daily activities?
3. Were there any specific questions in the EMA survey that you found difficult to answer or understand? If so, which ones and why?
4. Did you have any concerns about privacy when answering the EMA questions, especially those related to your social network and interactions with others?
5. How did you feel about wearing the accelerometer? Did it cause any discomfort or inconvenience?
6. Did participating in this study and answering questions about your social network and built environment change the way you thought about your physical activity or your surroundings? If so, how?
7. On a scale from 1 to 5, with 1 being very difficult and 5 being very easy, how would you rate the ease of use of the EMA app?
    - What made it easy or difficult to use?
    - Were there any technical issues you encountered while using the app?
8. On a scale from 1 to 5, with 1 being very uncomfortable and 5 being very comfortable, how would you rate your comfort level with answering questions about your social network and built environment in the moment?
    - What made you feel comfortable or uncomfortable about answering these questions?
9. If you could change anything about the study protocol (EMA frequency, accelerometer wear, types of questions asked), what would you change and why?
10. Would you be willing to participate in a similar study in the future? Why or why not?
